# Low frequency of N-methyl-D-aspartate receptor autoimmunity in tick-borne encephalitis

**DOI:** 10.1371/journal.pone.0334438

**Published:** 2025-10-13

**Authors:** Jakob Morén, Barbro Persson, Anna Sörman, Åke Lundkvist, Hanin Shihab, Marie Studahl, Malin Veje, Göran Günther, Gabriel Westman

**Affiliations:** 1 Infection Medicine, Department of Medical Sciences, Uppsala University, Uppsala, Sweden; 2 Infectious Diseases, Uppsala University Hospital, Uppsala, Sweden; 3 Department of Immunology, Genetics and Pathology, Uppsala University, Uppsala, Sweden; 4 Zoonosis Science Center, Department of Medical Biochemistry and Microbiology, Uppsala University, Uppsala, Sweden; 5 Department of Infectious Diseases, Institute of Biomedicine, Sahlgrenska Academy, University of Gothenburg, Gothenburg, Sweden; 6 Department of Infectious Diseases, Sahlgrenska University Hospital, Region Västra Götaland, Gothenburg, Sweden; Cleveland Clinic, UNITED STATES OF AMERICA

## Abstract

**Background:**

Tick-borne encephalitis is a viral infection of the central nervous system that may cause severe illness and long-term sequelae, to which underlying mechanisms are not completely understood. Autoantibodies against the N-methyl-D-aspartate receptor (anti-NMDAR) may be triggered by immunologic events, occur sporadically, and can cause autoimmune encephalitis. Following herpes simplex encephalitis and Japanese encephalitis, anti-NMDAR autoantibodies may develop and have been associated with relapse or impaired cognitive recovery. Tick-borne encephalitis has been shown to trigger anti-NMDAR encephalitis in sporadic cases, but the frequency of autoimmunization is unknown.

**Objectives:**

The objective of this study was to assess the frequency of intrathecal anti-NMDAR antibody development following tick-borne encephalitis and to explore whether such antibodies could be relevant to cognitive complaints.

**Methods:**

Adult patients with tick-borne encephalitis were included retrospectively from one cohort and prospectively from another. A stored post-acute cerebrospinal fluid sample was required for anti-NMDAR analysis. Two commercial kits (Euroimmun AG, Lübeck, Germany) were used to detect anti-NMDAR IgG antibodies in cerebrospinal fluid.

**Results:**

A total of 71 cerebrospinal fluid samples from 53 patients were analyzed for anti-NMDAR antibodies. Samples were obtained at a median of 91 days (range 21–471) after onset of central nervous system symptoms. Anti-NMDAR antibodies were detected in two samples from a single tick-borne encephalitis patient, corresponding to 1.9% of patients (95% CI: 0.05–10.1%).

**Conclusions:**

The development of intrathecal anti-NMDAR autoantibodies following tick-borne encephalitis is a rare event, and further studies are needed to clarify their potential relevance to cognitive outcomes in a minority of cases. Testing for anti-NMDAR antibodies in cerebrospinal fluid may be considered in patients who experience clinical deterioration following an initial recovery.

## Introduction

Tick-borne encephalitis (TBE) may lead to severe central nervous system (CNS) infection and frequently results in long-term sequelae, yet the underlying mechanisms remain poorly understood.

TBE is caused by the tick-borne encephalitis virus, an RNA virus belonging to the genus *Orthoflavivirus* (previously *Flavivirus*) within the family *Flaviviridae* [1–3]. Acute TBE typically manifests as a biphasic disease, reflecting its underlying pathogenesis. Following a tick bite and viral replication in draining lymph nodes, viremia occurs, leading to an initial phase characterized by fever and general symptoms. In a minority of infected individuals, viral neuroinvasion leads to a second phase of CNS infection, characterized by symptoms of meningoencephalitis, with or without myelitis. Clinical symptoms include headache, nausea, photophobia, neck stiffness, altered consciousness, loss of orientation, ataxia, tremor, dysphasia and spinal or cranial nerve paralysis. These symptoms are in contrast to the first viremic phase, which typically presents with nonspecific flu-like symptoms such as fever, malaise, myalgia, and fatigue. Approximately 18% of TBE patients have abnormal findings on brain magnetic resonance imaging (MRI), most often affecting the thalamus, nucleus caudatus, cerebellum and brainstem [1,2,4,5]. Unfortunately, there is no specific treatment for acute TBE infection; therefore, management focuses on supportive care [1,2,4,6].

The disease burden of TBE is primarily driven by long-term neurocognitive symptoms [2], with patients experiencing sequelae such as headaches, memory impairment, fatigue, and sensory or motor deficits [1,7]. The reported rate of sequelae varies depending on factors such as follow-up duration, study design, and loss to follow-up. However, approximately 35% of adult patients with meningoencephalitis report sequelae 12–18 months after infection [1,7–9], with residual symptoms potentially persisting for several years [8,10]. Thus, neurorehabilitation plays an important role in helping patients manage persistent symptoms [2].

Autoantibodies targeting the N-methyl-D-aspartate receptor (anti-NMDAR) were originally discovered in women with ovarian teratomas and paraneoplastic encephalitis [11]. Further research has shown that anti-NMDAR autoantibodies, whether triggered by an immunologic event such as cancer or infection, or occurring sporadically, can cause autoimmune encephalitis [12,13]. Clinically manifest anti-NMDAR encephalitis should be treated with immunomodulatory therapy [14]. In herpes simplex encephalitis (HSE), another severe virus infection in the CNS, anti-NMDAR frequently develop and are associated with an increased pro-inflammatory response, clinical relapse of encephalitis and possibly also sub-clinical impaired recovery of cognitive performance [15–17]. Other infections, including Japanese encephalitis (JE) caused by another *Orthoflavivirus*, have also been associated with the development of anti-NMDAR encephalitis, particularly in children [18–20].

Both innate and adaptive immune responses play important roles in TBE, with immunopathological mechanisms implicated in contributing to the disease [2]. In one study, TBE was shown to induce a stronger intrathecal IgG synthesis, reflected by the IgG index, compared to aseptic meningoencephalitis caused by enterovirus, herpes simplex virus type 2, or of unknown origin [7]. Two case reports have described TBE triggering the development of anti-NMDAR encephalitis [18,21]. A pilot study investigating anti-NMDAR and other antineuronal antibodies in serum from 22 selected TBE patients found no positive cases [22]. Although detection of anti-NMDAR antibodies in cerebrospinal fluid (CSF) has been shown to be more sensitive than serum testing for the diagnosis of anti-NMDAR encephalitis [23], the prevalence of such antibodies in CSF has not been systematically investigated in patients with TBE. This raises the question of whether the development of intrathecal anti-NMDAR autoantibodies could contribute to the high prevalence of cognitive sequelae observed following TBE.

### Objective

The objective of this study was to assess the frequency of intrathecal anti-NMDAR antibody development following TBE and to explore whether our findings provided support for the hypothesis that such antibodies may be relevant to cognitive complaints.

## Materials and methods

### Participants

Adult patients with TBE were included from two cohorts. Enrollment in the first cohort occurred between June 1991 and December 1993 at two study sites in Stockholm, Sweden: Danderyd Hospital and Huddinge Hospital. Patients were retrospectively included from this cohort, which was originally established for a prospective study on the clinical course and outcomes of meningoencephalitis of various etiologies [7]. Data from this cohort were accessed for research purposes on 27 May 2022. The second cohort consisted of prospectively included TBE patients enrolled at Uppsala University Hospital, Uppsala, and Sahlgrenska University Hospital, Gothenburg, Sweden, between 20 August 2021 and 1 February 2024.

For both cohorts, the virological diagnosis of TBE was established through the detection of specific IgM and IgG antibodies in serum, or alternatively, by demonstrating seroconversion or a four-fold increase in TBE-specific IgG antibodies in paired serum samples. In the second cohort, diagnosis was also established based on the detection of TBE-specific IgM in serum or CSF only, thereby including both confirmed and probable cases according to case definitions developed by the European Centre for Disease Prevention and Control [24]. All patients had symptoms of inflammation of the CNS, such as meningitis, meningoencephalitis or encephalomyelitis, and an elevated total leukocyte count in CSF (total CSF leukocyte count >5 × 10^6^ cells/L) at the time of diagnosis. At least one stored CSF sample obtained after the acute phase of CNS infection was required for the analysis of anti-NMDAR in this study.

### Follow-up and CSF sampling

Based on the clinical presentation, the acute disease was categorized as meningitis only, or as more complex disease with monofocal or multifocal neurological symptoms, including encephalitis. Patients were followed up with a clinical visit 1–3 months after the onset of CNS symptoms, at which point they were classified as either recovered or symptomatic based on self-reported persistent symptoms and objective findings. Symptoms such as memory impairment, concentration difficulties, mental fatigue, and reduced information processing were categorized as cognitive complaints. Objective cognitive testing was not performed systematically in all patients. However, if the examining clinician noted cognitive problems after any form of cognitive testing, the patient was classified as having cognitive complaints.

CSF was collected by lumbar puncture at the follow-up visit taking place 1–3 months after the onset of CNS symptoms. For some patients, a CSF sample was also collected approximately one year after the onset of CNS symptoms. All samples were aliquoted and frozen immediately after collection. Retrospectively included samples were initially stored at –20 °C, but later transferred to –80 °C. Long-term storage of all samples was at –80 °C. All transportation was carried out on dry ice, and freeze–thaw cycles were avoided.

### Neuronal antibody detection

Anti-NMDAR IgG were detected using two different commercially available kits (details provided below). Samples were classified as positive or negative based on the intensity of surface immunofluorescence of transfected cells compared to non-transfected cells. The tests were performed according to the manufacturer’s instructions. Slides were incubated with 30 µl undiluted CSF as previously described [25].

Detection of neuronal IgG autoantibodies was performed using a fixed cell-based assay with indirect immunofluorescence, employing either the BIOCHIP Mosaic 6 kit or glutamate receptor (type NMDA) kit (Euroimmun AG, Lübeck, Germany), both commercially available test systems. Slides are constructed with EU 90 cells, individually transfected with glutamate receptor (type NMDA), contactin-associated protein 2 (CASPR2), glutamate receptors (type AMPA1/2), leucine-rich glioma-inactivated protein 1 (LGI1), dipeptidyl aminopeptidase-like protein 6 (DPPX) and GABA B receptor and brought together as 6 BIOCHIP per test field (Mosaic 6 kit) or only NMDAR, 2 BIOCHIP per test field.

Side-by-side cells transfected with empty plasmids were fitted for negative control.

A positive patient control was included in all analyses. All slides were evaluated with a Nikon Eclipse E600 fluorescence microscope fitted with a LED lamp and all samples were blinded and independently evaluated by two analysts (BP and AS).

### Statistical analysis

Descriptive statistical analyses were conducted using R (version 4.3.0) [26] and RStudio (version 2023.06.0) [27], along with the R packages *binom*, *dplyr*, *ggplot2* and *vtable* [28–31]. Confidence intervals for proportions were estimated using the exact Clopper–Pearson method.

### Ethics statement

The initial study that established the first cohort was approved by the Ethical Committee of Karolinska University Hospital, Stockholm, (Reference No. 91:101) and included patients who provided informed consent. To allow further bioanalyses, all data and samples were fully anonymized, in line with an advisory opinion issued by the Ethics Review Appeals Board in Sweden (Reference No. Ö 75–2020/3.1). Thus, the authors had no access to any information that could identify individual patients, either during or after data collection. For the second cohort, ethical approval was obtained from the Swedish Ethical Review Authority (Reference No.2019−04711 and 2022-03954-02). Written informed consent was obtained from all patients in this cohort.

## Results

### Participants

A total of 53 TBE patients, aged 18–65 years (median: 45 years), with a CSF sample eligible for anti-NMDAR analysis were included. Clinical characteristics of the study participants are presented in Table 1.

**Table 1:** Clinical characteristics.

Variable	N	Percent
Sex	53	
Female	25	47%
Male	28	53%
Clinical presentation	53	
Meningitis only	20	38%
Encephalitis/other^a^	33	62%
Status at follow-up^b^	53	
Recovered	15	28%
Symptomatic	38	72%
Cognitive complaints at follow-up^b^	53	
No	23	43%
Yes	30	57%

^a^More complex disease with monofocal or multifocal neurological symptoms, including encephalitis.

^b^Assessed at 1–3 months after onset of CNS symptoms.

### CSF samples

**Fig 1:**
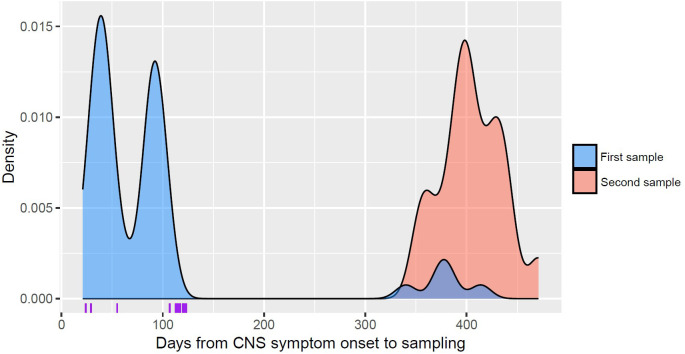
Days from CNS symptom onset to sampling. Density plot illustrating the time in days from onset of CNS symptoms to sample collection in TBE patients, separated into first samples (blue) and follow-up samples collected later from the same individuals (red). External HSE control data [25] are given in purple on the x-axis, illustrating the time from onset of CNS symptoms to the first CSF sample testing positive for anti-NMDAR.

A total of 71 CSF samples were analyzed for anti-NMDAR, with 21 collected from prospectively included patients and 50 from retrospectively included patients. The median time from onset of CNS symptoms to sample collection was 91 days (range 21–471 days). Fig 1 presents a density plot of the time to sample collection, separated into first and second samples (repeat samples from the same individual) collected during follow-up. For comparison, data from the first CSF sample that tested positive for anti-NMDAR IgG in each individual (n = 14) from a previous cohort of HSE patients [25] have been included.

### Anti-NMDAR

Two samples collected at 27 and 433 days after the onset of CNS symptoms from a single TBE patient, out of 53, tested positive for anti-NMDAR. Consequently, the estimated proportion of patients positive for anti-NMDAR autoantibodies during follow-up was 1.9% (95% confidence interval: 0.05–10.1%).

## Discussion

This study is, to our knowledge, the first to investigate the occurrence of intrathecal anti-NMDAR autoantibodies in a cohort of patients following TBE. Our main finding is that anti-NMDAR autoantibodies were detected in one patient following TBE, corresponding to a proportion of 1.9% (95% confidence interval: 0.05–10.1%). Given the high frequency of cognitive complaints after TBE and the low proportion of anti-NMDAR positivity observed, other mechanisms are likely to explain the majority of cognitive sequelae. Nevertheless, we cannot exclude the possibility that anti-NMDAR autoantibodies may be relevant to impaired recovery in rare cases.

Autoimmune encephalitis can occur sporadically or following an immunologic event, such as cancer or infection. In the specific context of anti-NMDAR encephalitis following infections, it is primarily associated with HSE [13]; however, case reports have also documented anti-NMDAR encephalitis following TBE and other meningoencefalitides including JE [18–21]. Following HSE, development of anti-NMDAR is associated with clinical relapse [15,16] and may also be related to sub-clinical impaired cognitive recovery [17]. HSE induces a robust immune response combined with neuronal cell death. It can be hypothesized that this process triggers autoimmunity and the subsequent development of anti-NMDAR autoantibodies [13,15,32]. The immune response induced by TBE is milder than that observed in HSE and appears to result in less brain injury. This is reflected by lower levels of neuronal and astroglial injury markers during the acute phase of TBE [17,33,34], although these marker levels tend to increase with disease severity [34]. Fewer abnormal findings on brain MRI have also been reported in TBE patients [5,35]. Assuming that the development of anti-NMDAR autoantibodies involves a combination of lytic infection and inflammation, this may help explain the lower proportion of positive patients following TBE compared to HSE.

The patient with samples positive for anti-NMDAR was a 27-year-old female who presented with biphasic TBE following a tick bite during the summer. The acute manifestation of TBE was meningitis. The patient was not hospitalized but was placed on sick leave for approximately six weeks. A follow-up visit, including CSF sampling, took place 27 days after the onset of CNS symptoms. During this visit, the patient reported persistent symptoms of tremor and headache. An additional CSF sample was collected 433 days after the onset of CNS symptoms, and both CSF samples tested positive for anti-NMDAR. At 433 days, the patient reported no remaining symptoms; however, neuropsychological evaluation indicated mild cognitive impairment. Unfortunately, a pre-TBE sample was not available from this patient for analysis of anti-NMDAR.

Our findings indicate that the development of anti-NMDAR autoantibodies does not account for the high prevalence of cognitive complaints observed following TBE. Nevertheless, we cannot exclude that these antibodies might be relevant for cognitive recovery after TBE in rare cases. Interestingly, the patient who tested positive for anti-NMDAR reported no symptoms when the last CSF sample was collected, even though neuropsychological evaluation indicated mild cognitive impairment. It is possible that the patient had residual sequelae, which she was either unaware of or did not recognize as symptoms, and that these sequelae were influenced by the presence of anti-NMDAR. Since no pre-TBE baseline sample was available for this patient, we were unable to confirm whether the development of anti-NMDAR was triggered by TBE. Therefore, another possibility is that the anti-NMDAR autoantibodies were not triggered by TBE. However, this appears unlikely given the very low incidence of anti-NMDAR encephalitis [36] as well as the low incidence of TBE [37], and the fact that anti-NMDAR antibodies in CSF are typically not detected in control populations [12,23,38,39]. It is also possible that the anti-NMDAR autoantibodies, regardless of whether they were triggered by TBE, had no significant impact on the outcome in this case. Furthermore, both the previously reported cases of anti-NMDAR encephalitis following TBE [18,21] and the patient who tested positive for anti-NMDAR in this study had relatively mild acute disease. This suggests that the development of anti-NMDAR autoantibodies can be immunologically triggered even in the absence of extensive cell death in the CNS.

A strength of this study is that a total of 71 CSF samples from 53 TBE patients, with a typical frequency of cognitive complaints at follow-up, were analyzed for anti-NMDAR antibodies.

The study also has several limitations. One is the limited availability of CSF samples from the full study cohorts, which may have introduced a selection bias in our results. For the samples collected between 1991 and 1993, another limitation is the long-term storage of samples, spanning several decades, which could potentially affect the results. However, we consider antibodies to be stable and unlikely to degrade significantly in frozen samples. Data from the previous study on anti-NMDAR autoantibodies in HSE patients [25] support the notion that these antibodies remain stable during long-term storage under similar conditions. The stability of anti-NMDAR antibodies in frozen samples is further supported by the fact that the samples testing positive for anti-NMDAR in our study were collected in 1992 and 1993. Additionally, the time from the onset of CNS infection to sampling, which started at 21 days, may be considered relatively short for some of the analyzed samples in the context of autoantibody development. This could potentially result in the misclassification of patients with a sample collected early during follow-up. However, in case reports of anti-NMDAR encephalitis following TBE patients were positive for anti-NMDAR after one month and 26 days, respectively [18,21]. In a previous cohort of HSE patients [25], the median time from onset of CNS symptoms to the detection of anti-NMDAR autoantibodies was 115 days (range: 24–123 days), as illustrated in Fig 1. However, the time to detection was influenced by the timing of CSF sample collection according to the study protocol. In the study by Prüss et al. [15], the earliest CSF samples from individuals who tested positive for anti-NMDAR IgG were collected at 5, 9, and 30 days after the onset of HSE. Furthermore, the latest positive CSF samples were collected at 121, 2,225, and 3,268 days, suggesting a broad interval of positivity. All this, together with the fact that the patient with anti-NMDAR tested positive on day 27, suggests that clinically relevant anti-NMDAR autoantibodies can be detected within the interval for sample collection in this study. Finally, a limitation is the lack of pre-TBE baseline sample for the patient positive for anti-NMDAR.

## Conclusions

Intrathecal anti-NMDAR autoantibodies appear to be a rare finding after TBE and does not explain the high frequency of cognitive complaints. In a broader perspective, it cannot be excluded that these antibodies may be of relevance for cognitive outcomes in a minority of cases, but further studies are needed to clarify their role. Clinically, this suggests that testing for anti-NMDAR antibodies in CSF may be considered in patients with cognitive impairment following TBE, particularly if there are signs of clinical deterioration following an early recovery, as immunomodulatory therapy should be considered for anti-NMDAR positive patients.

## Supporting information

S1 Dataset Clinical characteristics of patients. (CSV)

S2 Dataset Sample information and anti-NMDAR results. (CSV)

S3 Dataset HSE comparison data. (CSV)
